# Progress in research on the association between glucose metabolism disorders and intracranial and extracranial atherosclerotic stenosis

**DOI:** 10.3389/fcvm.2026.1866635

**Published:** 2026-06-08

**Authors:** Mingyan Zhang, Huiyong Huo, Rui Yang, Yunao Gao, Yibo Jia, Juntao Li

**Affiliations:** 1 Hebei North University, Zhangjiakou, Hebei, China; 2 The Central Hospital of Handan, Handan, Hebei, China; 3 Hebei Medical University, Shijiazhuang, Hebei, China; 4 Chengde Medical University, Chengde, Hebei, China; 5 The First Hospital of Handan, Handan, Hebei, China

**Keywords:** diabetes, extracranial atherosclerosis, glucose metabolism disorders, intracranial atherosclerosis, stroke

## Abstract

**Objective:**

Intracranial atherosclerotic stenosis (ICAS) and extracranial atherosclerotic stenosis (ECAS) are major vascular substrates for ischemic stroke. Existing reviews often discuss ICAS and ECAS together, overlooking their distinct pathological responses to glucose dysregulation, and lack systematic summary of the independent role of insulin resistance in normoglycemic individuals. This review aims to systematically summarize the molecular mechanisms, imaging features, clinical biomarkers, and intervention strategies by which glucose metabolism disorders (diabetes mellitus, insulin resistance, impaired glucose tolerance) promote ICAS and ECAS.

**Methods:**

This is a narrative review of relevant literature published in recent years, with no systematic meta-analysis performed.

**Results:**

Hyperglycemia and insulin resistance synergistically accelerate atherosclerosis through accumulation of advanced glycation end products (AGEs), enhanced oxidative stress, activation of the NOD-like receptor thermal protein domain associated protein 3 (NLRP3) inflammasome, and phenotypic switching of vascular smooth muscle cells (VSMCs). Clinical studies have confirmed that hemoglobin A1c (HbA1c) ≥ 6.5% is positively correlated with anterior circulation ICAS (OR = 2.04, *P* < 0.05). The triglyceride-glucose (TyG) index is significantly positively correlated with asymptomatic ECAS (OR = 1.85) and asymptomatic ICAS (OR = 1.34). The 30-year follow-up of the Da Qing Diabetes Prevention Study showed that individuals with impaired glucose tolerance who maintained non-diabetic status through intervention had a 23% lower risk of stroke compared to those with newly diagnosed diabetes (HR = 0.77, 95%CI 0.64–0.94).

**Conclusion:**

Hyperglycemia and insulin resistance accelerate intracranial and extracranial atherosclerosis via AGEs accumulation, oxidative stress, NLRP3 inflammasome activation, and VSMC phenotypic switching. HbA1c and the TyG index can serve as clinical screening tools. Key questions remain regarding whether different subtypes of glucose metabolism disorders differentially affect intracranial versus extracranial arteries, and whether early intervention in prediabetic populations reduces stroke risk. This review provides a theoretical reference for risk stratification and individualized prevention in populations at high risk of ischemic stroke.

## 1 Introduction

Ischemic stroke is the leading cause of disability and death among Chinese residents, and atherosclerotic stenosis is the most direct driver of this disease (1). Based on the location of stenosis, it can be divided into intracranial atherosclerotic stenosis (ICAS) and extracranial atherosclerotic stenosis (ECAS). The population distributions of these two types of stenosis differ significantly: ICAS is more common in Asian populations, whereas ECAS predominates in European and American populations (2, 3). This difference suggests that, in addition to traditional risk factors (hypertension, hyperlipidemia, etc.), genetic background and metabolic status also play important roles (4).

Over the past two decades, the widespread epidemic of obesity and metabolic syndrome has expanded the impact of glucose metabolism disorders beyond the boundaries of traditional endocrine diseases. A large body of evidence indicates that not only clinically diagnosed diabetes, but also impaired fasting glucose, impaired glucose tolerance, and even isolated insulin resistance are clearly associated with an increased risk of atherosclerotic cardiovascular events (5). Of particular note, the risk of ischemic stroke in patients with diabetes is approximately 1.5–2 times that of non-diabetic individuals (6, 7); moreover, structural changes in the arterial wall can already be observed during the “prediabetic” stage when diagnostic criteria for diabetes have not yet been met.

It must be pointed out that in most published reviews on glucose metabolism disorders and atherosclerosis, ICAS and ECAS are not discussed separately but are uniformly grouped under the concept of “intracranial and extracranial atherosclerosis.” This actually implies an assumption that the two types of vessels have the same response pattern to glucose dysregulation. However, from an anatomical perspective, intracranial arteries lack an external elastic lamina, have few vasa vasorum in the adventitia, and are chronically exposed to higher blood flow shear stress; extracranial arteries have thicker vessel walls and a richer supply of vasa vasorum in the adventitia (8). These structural differences suggest that the sensitivity of the two to glucose metabolism abnormalities may be intrinsically different (9) (Table 1). Furthermore, most reviews focus on patients with clinically diagnosed diabetes, whereas for the large population with completely normal blood glucose levels but existing insulin resistance, whether vascular damage is independent of hyperglycemia and to what extent damage occurs have not been systematically summarized.

**Table 1:** Comparison of characteristics between intracranial and extracranial atherosclerotic stenosis.

Feature	Intracranial Arterial Stenosis (ICAS)	Extracranial Arterial Stenosis (ECAS)
Predilection site	Middle cerebral artery, intracranial internal carotid artery	Carotid bifurcation, initial segment of internal carotid artery
Vessel wall structure	Thin, poorly developed external elastic lamina	Thick, intact external elastic lamina
Pathological characteristics	Predominantly diffuse intimal thickening	Eccentric lipid-rich plaque, prone to calcification and hemorrhage
Racial difference	More common in Asian populations	More common in European and American populations

Although many studies have explored the relationship between glucose metabolism disorders and atherosclerosis, three questions remain that have not been systematically addressed in reviews. First, most studies analyze ICAS and ECAS together, ignoring differences in vessel wall structure, hemodynamics, and susceptibility to metabolic factors. Second, whether insulin resistance independently contributes to intracranial and extracranial arterial stenosis in people with completely normal blood glucose levels lacks integrated evidence. Third, clinical intervention evidence comes almost exclusively from diabetic patients, and whether it can be extrapolated to the much larger prediabetic population remains inconclusive.

Based on the above background, this review aims to summarize the main molecular pathways by which glucose metabolism disorders (diabetes, insulin resistance, impaired glucose tolerance) promote atherosclerosis; to summarize new findings from clinical imaging and biomarker studies on intracranial and extracranial arterial stenosis; and to highlight current controversies and possible future research directions, providing a clearer reference for the early prevention of ischemic stroke.

## 2 Methods

This review is a narrative review, not a systematic review. We searched PubMed, Web of Science for articles published from January 2015 to December 2025. The search keywords included combinations of: “glucose metabolism disorders”, “diabetes mellitus”, “insulin resistance”, “impaired glucose tolerance”, “intracranial atherosclerosis”, “extracranial atherosclerosis”, “ICAS”, “ECAS”, “stroke”, “TyG index”, and “HbA1c”. We also manually screened the reference lists of included articles. Only English or Chinese full-text articles were considered. Conference abstracts, case reports, and editorials were excluded.

## 3 Main types of glucose metabolism disorders and their vascular pathological significance

Glucose metabolism disorders are not a single entity; different subtypes differ in blood glucose fluctuation patterns, insulin secretion and action status, and each has distinct characteristics in terms of vascular injury pathways.

### 3.1 Diabetes mellitus

Diabetes is the most typical and well-studied type of glucose metabolism disorder, characterized by chronic long-term hyperglycemia. Numerous animal experiments and human pathological studies have confirmed that hyperglycemia damages blood vessels through at least three pathways: First, non-enzymatic reaction of glucose with proteins generates advanced glycation end products (AGEs), which bind to the receptor for advanced glycation end products (RAGE) on the vessel wall and initiate an inflammatory cascade (10, 11); second, hyperglycemia activates the polyol pathway and protein kinase C (PKC) pathway, leading to enhanced intracellular oxidative stress and reduced bioavailability of nitric oxide (NO) (12); third, hyperglycemia can directly damage tight junctions between endothelial cells and increase vascular permeability (13).

One relevant study included 360 patients who underwent DSA and showed that ICAS severity increased with HbA1c level; specifically, HbA1c ≥ 6.5% was significantly positively correlated with anterior circulation stenosis (*r* = 0.13, *P* < 0.05), and a positive trend was also observed for posterior circulation stenosis (*r* = 0.13, *P* > 0.05). Further analysis indicated that in patients aged > 68 years with elevated HbA1c, the odds ratio for anterior circulation ICAS was 2.04 (95%CI: 1.11–3.81, *P* < 0.05), whereas in those aged ≤ 68 years, the odds ratio for posterior circulation ICAS was 2.12 (95%CI: 1.16–3.97, *P* < 0.05), directly demonstrating a dose-response relationship between hyperglycemia and ICAS severity (14).

### 3.2 Insulin resistance

The concept of insulin resistance is broader than that of diabetes. Some individuals have normal blood glucose test results, but the sensitivity of muscle, fat, and liver tissues to insulin has already decreased. The effect of insulin resistance on blood vessels is bidirectional. Under physiological conditions, insulin promotes NO production through the phosphatidylinositol 3-kinase (PI3K)/protein kinase B (Akt) pathway, causing vasodilation; simultaneously, it releases endothelin-1 through the mitogen-activated protein kinase (MAPK) pathway, causing vasoconstriction (15, 16). Normally, the two are in balance. In insulin-resistant states, the PI3K/Akt pathway is impaired while the MAPK pathway is relatively hyperactive, resulting in predominance of vasoconstriction and impaired endothelial function (17). This mechanism explains why some individuals with “normal blood glucose” still experience atherosclerotic events.

One large prospective cohort study including 430,093 non-diabetic individuals showed, after a median follow-up of 13.5 years, that insulin resistance level assessed by estimated glucose disposal rate (eGDR) was significantly associated with stroke risk in a non-linear dose-response manner; when eGDR was above 7.64, each unit increase was associated with a 20% lower risk of stroke (HR = 0.80, 95% CI: 0.78–0.82), and this effect was consistent for both ischemic and hemorrhagic stroke (18). This study provides evidence-based dose-response evidence supporting that improving insulin sensitivity may be an independent vascular protective strategy.

### 3.3 Impaired glucose tolerance

Impaired glucose tolerance (IGT) is an intermediate state between normal glucose and diabetes, characterized by normal fasting blood glucose but elevated postprandial blood glucose. A cross-sectional study showed that in patients with Pre-IGT (an earlier stage of IGT), 21.74% already had decreased flow-mediated dilation (FMD), and elevated serum insulin and HbA1c levels were significantly negatively correlated with FMD values (*P* < 0.05) (19). This suggests that vascular injury begins at an even earlier stage of prediabetes, which is difficult to capture by conventional risk assessment. Two latest analyses of the Da Qing Diabetes Prevention Study based on 30-year follow-up further confirmed that if people with IGT return to and maintain a non-diabetic status through intervention, their long-term risk of cardiovascular events and stroke is significantly reduced, with stroke risk decreased by 23% compared to newly diagnosed diabetic patients (HR = 0.77, 95% CI: 0.64–0.94), and maintaining a non-diabetic status for at least 4 years yields significant survival benefits (20, 21). Therefore, the intervention window for atherosclerosis should be moved forward to the prediabetic stage, rather than waiting until the diagnostic criteria for diabetes are met.

## 4 Main mechanisms by which glucose metabolism disorders promote atherosclerosis

The damage of glucose metabolism disorders to the arterial wall is not a single pathway but a complex network of multiple interacting factors. The following four mechanisms are among the most strongly supported.

### 4.1 Advanced glycation end products (AGEs) accumulation

Hyperglycemia promotes the formation of AGEs, which bind to RAGE on endothelial cells, activate NF-κB, and upregulate adhesion molecules (VCAM-1, ICAM-1), thereby recruiting inflammatory cells (22). In insulin-resistant states, hyperinsulinemia can also upregulate RAGE expression and enhance AGE effects. AGEs also cross-link collagen and elastin, increasing vessel stiffness (23).

### 4.2 Positive feedback loop of oxidative stress

High glucose increases mitochondrial superoxide production, leading to excessive reactive oxygen species (ROS) that directly damage endothelial cells and promote low-density lipoprotein oxidation into ox-LDL (23). Macrophages take up ox-LDL and transform into foam cells, while ox-LDL itself further induces ROS generation, creating a self-reinforcing positive feedback loop (24).

### 4.3 NLRP3 inflammasome and persistent inflammation

High glucose and elevated ROS activate the NLRP3 inflammasome in macrophages, promoting maturation and release of IL-1β and IL-18, thereby recruiting more immune cells into plaques (25, 26).Activated macrophages secrete matrix metalloproteinases (MMPs) that degrade collagen and elastin in the fibrous cap, and inhibiting MMP8 has been shown to increase fibrous cap thickness and reduce intraplaque hemorrhage (27). This establishes a causal chain from inflammation to plaque instability.

### 4.4 Phenotypic switching of vascular smooth muscle cells

Vascular smooth muscle cells (VSMCs) normally exhibit a contractile phenotype but can switch to a synthetic phenotype under pathological stimulation, migrating into the intima and secreting extracellular matrix (28, 29). In high-glucose and insulin-resistant environments, this switching is skewed toward a pathological direction: high glucose promotes VSMC proliferation and increases mitochondrial calcium uptake, leading to metabolic reprogramming (30). After vascular injury, the neointimal area is larger in diabetic mice, and knockout of specific transcription factors such as Twist1 partially reverses this effect (31, 32).

### 4.5 Synergistic effects of lipid metabolism disorders

Glucose metabolism disorders are often accompanied by lipid metabolism disorders. In insulin-resistant states, adipose tissue lipolysis is enhanced, free fatty acid release increases, and the liver synthesizes more very low-density lipoprotein (VLDL), leading to elevated triglycerides (33). At the same time, the proportion of small dense low-density lipoprotein (sd-LDL) increases, and high-density lipoprotein (HDL) function is impaired. Current views suggest that sd-LDL readily enters the arterial wall and becomes oxidized, while dysfunctional HDL loses its ability to promote reverse cholesterol transport; the two synergistically accelerate atherosclerosis progression (34). In clinical practice, the triglyceride-glucose (TyG) index is a simple tool that integrates fasting blood glucose and triglycerides to indirectly assess insulin resistance and atherosclerotic burden (35).

## 5 Clinical studies on glucose metabolism disorders and intracranial/extracranial arterial stenosis

In this section, we summarize clinical evidence from cross-sectional, prospective, and imaging studies that have separately evaluated the association between glucose metabolism disorders and intracranial atherosclerotic stenosis (ICAS) versus extracranial atherosclerotic stenosis (ECAS). Emphasis is placed on differential effects observed with biomarkers such as the TyG index and HbA1c, as well as insights from high-resolution vessel wall MRI.

### 5.1 Cross-sectional and prospective studies

A retrospective study showed that in populations at high risk of stroke, those with a higher TyG index tended to have a higher detection rate of ICAS compared to those with a lower TyG index, although the exact effect size needs further quantification; similar trends were obtained for ECAS analysis (9). Additionally, a systematic review and meta-analysis summarized that the mean carotid intima-media thickness in prediabetic populations was higher than in normoglycemic populations (36). These data consistently indicate that the effect of glucose metabolism disorders on the arterial wall appears before blood glucose reaches the diagnostic criteria for diabetes. It is worth noting that the above findings are not entirely consistent. One community-based cohort study found that an elevated TyG index was significantly positively correlated with both prevalent and incident ECAS (OR = 1.34 and 1.85, respectively), but not significantly associated with ICAS (9). This result suggests that the insulin-resistant state reflected by the TyG index may have a more direct influence on extracranial arteries, whereas its association with intracranial arterial stenosis requires further validation in large-sample studies.

### 5.2 High-resolution vessel wall magnetic resonance imaging evidence

Advances in imaging technology have made it possible to observe the characteristics of atherosclerotic plaques *in vivo*. One study used high-resolution magnetic resonance vessel wall imaging to analyze 958 intracranial plaques from 225 patients with acute ischemic stroke. The results showed that patients with type 2 diabetes mellitus (T2DM) had a significantly greater number of intracranial plaques than non-T2DM patients (4.80 ± 2.22 vs. 3.60 ± 1.78, *P* < 0.05). Moreover, the enhancement ratio of symptomatic plaques in T2DM patients with poor glycemic control (2.32 ± 0.61) was significantly higher than that in patients with good glycemic control (1.60 ± 0.62) and non-diabetic patients (1.39 ± 0.39), with all differences statistically significant (all *P* < 0.05). Multivariate regression analysis showed that HbA1c level was independently associated with plaque enhancement ratio (beta = 0.641, *P* < 0.05). Plaque enhancement is generally considered an imaging marker of inflammation and neovascularization activity and is closely related to the risk of clinical events. In addition, the study found that the incidence of positive remodeling was higher in diabetic patients. Although positive remodeling can temporarily preserve luminal area, it is often accompanied by larger plaque volume and higher instability (37).

### 5.3 Clinical value of biomarkers

Currently, the most widely used glucose metabolism-related biomarkers in clinical practice include glycated hemoglobin (HbA1c) and the TyG index. A national cohort study found that the combination of TyG index and high-sensitivity C-reactive protein (hs-CRP) could better predict new-onset cardiovascular events, with a significantly increased risk in individuals with high levels of both (38). This result suggests that integrating inflammatory markers may improve risk stratification in populations with glucose metabolism disorders. Furthermore, recent studies have extended the prognostic value of the TyG index to stroke outcomes. For example, one study reported that elevated TyG index was independently associated with increased risk of recurrent stroke and poor functional outcome after ischemic stroke (39).

## 6 Intervention strategies

The aforementioned accumulation of AGEs, oxidative stress, NLRP3 inflammation, and VSMC phenotypic switching together constitute a pathological network through which glucose metabolism disorders promote atherosclerosis. Accordingly, intensive glycemic control seems the most direct intervention, but its clinical benefit is limited, suggesting that we need to go beyond “glucose-centric” thinking. This section reviews existing strategies from four aspects: glycemic control, comprehensive management, new antidiabetic drugs, and prediabetes intervention.

### 6.1 Goals and controversies of glycemic control

Long-term follow-up studies have confirmed that early intensive glycemic control reduces microvascular complications in type 2 diabetes, but its protective effect on macrovascular events takes a long time to emerge. However, some studies have shown that in patients with type 2 diabetes at high cardiovascular risk, too strict glycemic control (e.g., reducing HbA1c to very low levels) not only failed to reduce major cardiovascular events but might increase mortality, with hypoglycemic events considered an important reason (40). Based on the above evidence, current guidelines emphasize individualization of glycemic management: stricter targets may be used for younger patients with short disease duration and no severe complications, whereas targets should be appropriately relaxed for elderly patients, those with long disease duration, a history of severe hypoglycemia, or multiple comorbidities (41). In addition, increasing evidence in recent years has focused on glycemic variability, which may be more vascularly toxic than sustained hyperglycemia.

### 6.2 Comprehensive management of multiple risk factors

Glycemic control alone is insufficient to fully reduce the risk of atherosclerotic vascular events; comprehensive management of blood pressure, lipids, and antiplatelet therapy is required. For blood pressure management, current guidelines generally recommend that patients with atherosclerosis and diabetes achieve a relatively strict blood pressure target (42). Lipid management is based on statins, with LDL-cholesterol targets determined according to patient risk stratification; high-risk patients are advised to achieve lower levels, and very high-risk patients may consider even lower targets. Antiplatelet therapy requires individualized balancing of ischemic and bleeding risks; aspirin remains a first-line choice but is not recommended for routine use in diabetic patients without established cardiovascular disease (43). Lifestyle interventions (reasonable diet, regular exercise, smoking cessation, alcohol restriction, weight control) provide additional benefit beyond the above pharmacotherapies (44). Multiple studies have confirmed that comprehensive intervention on multiple risk factors in patients with type 2 diabetes significantly reduces cardiovascular events and all-cause mortality.

### 6.3 Cardiovascular protective evidence of new antidiabetic drugs

In recent years, two classes of new antidiabetic drugs, sodium-glucose cotransporter 2 (SGLT2) inhibitors and glucagon-like peptide-1 (GLP-1) receptor agonists, have demonstrated cardiovascular protective effects beyond glucose lowering in cardiovascular outcome trials (45). Clinical studies have shown that SGLT2 inhibitors (e.g., empagliflozin) significantly reduce the risk of major adverse cardiovascular events in patients with type 2 diabetes; this benefit appears shortly after treatment initiation and is not significantly correlated with the magnitude of HbA1c reduction (46). Similar cardiovascular protective effects have been observed for GLP-1 receptor agonists (e.g., liraglutide). Current views suggest that the cardiovascular protective mechanisms of these drugs involve multiple pathways, including improved endothelial function, inhibition of inflammation, reduction of oxidative stress, weight loss, and blood pressure lowering, rather than relying solely on glucose-lowering effects (47). For patients with type 2 diabetes complicated by atherosclerosis, major guidelines have clearly recommended preferential use of these two drug classes. Notably, accumulating evidence supports the role of these agents in stroke prevention. A recent study demonstrated that SGLT2 inhibitors were associated with lower risk of ischemic stroke in patients with type 2 diabetes (48). Similarly, GLP-1 receptor agonists have shown beneficial effects on cerebrovascular outcomes in high-risk populations (49).

### 6.4 Population-specific interventions targeting insulin resistance

Unlike patients with diagnosed diabetes, individuals with isolated insulin resistance (normal blood glucose) or impaired glucose tolerance have an increased risk of stroke, but the absolute risk level is lower (50). Currently, there is a lack of large-scale randomized controlled trials with cardiovascular or cerebrovascular endpoints for the use of SGLT2 inhibitors or GLP-1 receptor agonists in this population. Existing evidence suggests that lifestyle interventions (dietary modification, regular exercise) have a protective effect on vascular health in this population (51). Whether metformin independently reduces stroke risk in normoglycemic individuals with insulin resistance remains unclear; current evidence comes mainly from secondary or *post-hoc* analyses of diabetes prevention studies. Future priority should be given to designing early intervention trials in populations with normal blood glucose but high insulin resistance burden (e.g., elevated TyG index) to test the feasibility of blocking or delaying the atherosclerotic process while blood glucose is still in the normal range.

## 7 Discussion

Integrating the above evidence leads to three key insights. First, glucose metabolism disorders accelerate intracranial and extracranial atherosclerosis through at least four intersecting pathways (AGEs accumulation, oxidative stress, NLRP3 inflammasome activation, and VSMC phenotypic switching), and these pathways exhibit positive-feedback mutual amplification. Second, even when blood glucose levels are completely normal, insulin resistance alone can independently increase the risk of arterial stenosis and plaque vulnerability; HbA1c and TyG index can serve as convenient clinical screening tools. Third, high-resolution vessel wall magnetic resonance imaging studies show that patients with diabetes have more vulnerable intracranial and extracranial plaques, including higher enhancement degree, more frequent positive remodeling, and greater overall plaque burden.

However, several key disagreements and gaps remain in the existing literature. First, is there a difference in the sensitivity of intracranial and extracranial arteries to glucose metabolism disorders? Existing epidemiological data show a stronger association between ICAS and diabetes in Asian populations than between ECAS and diabetes in Caucasian populations, but this racial difference cannot distinguish genetic from metabolic factors (52, 53). Theoretically, intracranial arteries, due to the lack of an external elastic lamina and scarcity of adventitial vasa vasorum, may be less tolerant to metabolic inflammation and oxidative stress. However, to date, no prospective study has directly compared the progression rate or plaque vulnerability of ICAS and ECAS under the same metabolic state using identical imaging criteria (e.g., high-resolution vessel wall MRI). Addressing this issue will require designing multicenter cohorts with diverse ethnicities, strictly controlling for confounders such as blood glucose, blood pressure, and lipids, and performing head-to-head imaging follow-up. Second, the vast majority of intervention studies have been conducted in patients with type 2 diabetes; evidence regarding whether the same glycemic strategies are effective for macrovascular disease in type 1 diabetes is extremely scarce (54, 55). Third, although agents such as RAGE antagonists and PKC inhibitors have shown some potential in animal experiments, clinical translation has repeatedly failed, suggesting that single-target blockade may be ineffective due to pathway redundancy (56, 57).

Moreover, recent studies have gradually revealed the important role of epigenetic regulation in glucose metabolism disorder-related atherosclerosis (58). DNA methylation, histone modifications, and non-coding RNAs (e.g., miR-126, miR-155) can respond to high glucose and insulin resistance stimuli, regulating the expression of inflammatory factors in endothelial cells, transcription of oxidative stress-related genes, and phenotypic switching of VSMCs (59, 60). In particular, miR-126 maintains vascular integrity by targeting SPRED1; its decreased plasma level in diabetic patients is negatively correlated with carotid intima-media thickening, whereas miR-155 participates in plaque progression by activating endothelial-mesenchymal transition (61, 62). These findings suggest that epigenetic markers may become novel molecular markers for early screening of intracranial and extracranial arterial stenosis in the future, and drugs targeting epigenetic modifications (such as histone deacetylase inhibitors) may become new strategies for intervening in glucose metabolism disorder-related vascular damage, but they are still in the preclinical exploration stage.

Future research may achieve breakthroughs in the following directions. First, conduct large-scale, multicenter, ethnically diverse prospective cohort studies using uniform high-resolution imaging criteria to directly compare the natural history differences between ICAS and ECAS under conditions of glucose metabolism disorders. Second, design randomized controlled trials targeting prediabetic populations to assess whether early lifestyle intervention or metformin/SGLT2 inhibitors can delay the progression of intracranial and extracranial arterial stenosis. Third, explore multi-target combination anti-inflammatory or anti-oxidant strategies, because in the face of complex metabolic disorders, the single-drug, single-target intervention model may no longer meet clinical needs.

### 7.1 Limitations of this review

This review has several limitations. First, as a narrative review, it did not employ a systematic search protocol or formal quality assessment of included studies. Therefore, selection bias and incomplete retrieval of relevant literature cannot be excluded. Second, most of the cited studies are cross-sectional or retrospective, and prospective head-to-head comparisons between ICAS and ECAS under identical metabolic conditions are lacking. Third, the majority of evidence comes from Asian or Western populations separately, limiting the generalizability of ethnic comparisons.

### 7.2 Critical appraisal of the current evidence

The association between glucose metabolism disorders and arterial stenosis is supported by multiple studies, but the quality of evidence varies considerably. Many studies rely on single biomarkers (e.g., HbA1c or TyG) without adjusting for important confounders such as duration of diabetes, use of statins, or socioeconomic status. Furthermore, the definition of ICAS and ECAS is not uniform across imaging modalities (CTA, MRA, DSA), leading to potential heterogeneity. Future prospective cohort studies with standardized imaging protocols and blinded outcome adjudication are urgently needed.

## 8 Conclusion

The main novelties of this review are as follows:

(1) A core contribution of this review is to clearly distinguish the different pathological effects of glucose metabolism disorders on intracranial arteries versus extracranial arteries, rather than discussing them together. By comparing the vessel wall structure, predilection sites, and differential responses to metabolic factors between the two, it explicitly points out that future studies should use uniform imaging criteria to directly compare the progression rates of ICAS and ECAS.
(2) For the first time, it systematically summarizes the independent pathogenic role of insulin resistance in the normoglycemic stage, rather than focusing only on clinically diagnosed diabetes. Based on evidence from eGDR, TyG index, and others, it confirms that insulin resistance alone, even with completely normal blood glucose, can independently increase the risk of arterial stenosis and plaque vulnerability.
(3) Based on existing evidence, it clearly proposes a strategy of moving the intervention window forward to the prediabetic stage. Combined with the 30-year follow-up data of the Da Qing Diabetes Prevention Study, it points out that intervention for atherosclerosis should not wait until the diagnostic criteria for diabetes are met, but should be advanced to the stage of impaired glucose tolerance.

The two most important future directions are to clarify the differential effects of glucose metabolism subtypes on intracranial versus extracranial arteries, and to conduct randomized controlled trials of early intervention in prediabetic populations.
